# Supplementary material for: Intracellular Injection of Brain Extracts from Alzheimer’s Disease Patients Triggers Unregulated Ca2+ Release from Intracellular Stores That Hinders Cellular Bioenergetics
Source: Cells. 2022 Nov 16;11(22):3630. doi: 10.3390/cells11223630 (PMC9688564; doi:10.3390/cells11223630)
Supplement: Supplementary file 1 [file cells-11-03630-s001.zip › cells-1897942-supplementary.pdf]

cells

MDPI

Article

Intracellular Injection of Brain Extracts from Alzheimer's Disease Patients Triggers Unregulated Ca^{2+} Release from Intracellular Stores That Hinders Cellular Bioenergetics

Anna Pensalfini [1], Abdul Rahim Umar [2], Charles Glabe [1], Ian Parker [3,4], Ghanim Ullah [2,*] and Angelo Demuro [3,*]

Whole-cell Ca^{2+} signaling model

Extracts from AD-affected brains stimulate the production of IP_3 through PLC, which binds to IP_3Rs to release Ca^{2+} from the ER (J_{ipr}). Ca^{2+} is also released from the ER through leak channels (J_{leak}), pumped back into the ER through Sarco/ER Ca^{2+}-ATPase (SERCA) (J_s), and buffered by Ca^{2+} sensitive dye Fluo-4 ([dye]). Thus the rate equation for cytosolic Ca^{2+} concentration ($[Ca^{2+}]_i$) is given as

$$\frac{d[Ca^{2+}]_i}{dt} = J_{ipr} + J_{leak} - J_s + k^r_{dye}\left(B_{dye} - [dye]\right) - k^f_{dye}[Ca^{2+}]_i[dye].$$

Where B_{dye} is the total concentration of Fluo-4 and k^f_{dye} and k^r_{dye} are the binding and unbinding rates of Ca^{2+} to Fluo-4 respectively. Note that Ca^{2+} is also buffered by endogenous cytosolic buffers, which are not included in the model. Nevertheless, this does not change the conclusions from the model.

The functional form of the three fluxes are adopted from [1] and given as

$$J_{ipr} = k_{ipr}O([Ca^{2+}]_{ER} - [Ca^{2+}]_i),$$

$$J_{leak} = k_{leak}([Ca^{2+}]_{ER} - [Ca^{2+}]_i),$$

$$J_s = \frac{V_s[Ca^{2+}]_i^{ns}}{K_s^{ns} + [Ca^{2+}]_i^{ns}}.$$

Ca^{2+} concentration in the ER ($[Ca^{2+}]_{ER}$) is given by the conservation of total Ca^{2+} ($[Ca^{2+}]_t$) in the cell, i.e.

$$[Ca^{2+}]_{ER} = \gamma([Ca^{2+}]_t - [Ca^{2+}]_i).$$

Rate equation for Fluo-4 is given as

$$\frac{d[dye]}{dt} = k^r_{dye}\left(B_{dye} - [dye]\right) - k^f_{dye}[Ca^{2+}]_i[dye].$$

Parameter values used in the whole-cell Ca^{2+} model are given in [2].

To model the gating of IP_3R, we use our previously developed kinetic scheme that closely replicates the behavior of type 1 IP_3R in *Xenopus* laevis oocytes (see Fig. 2 in [3]). The model has four states: a rest state (*R*) with no Ca^{2+} bound, an active state (*A*) with 2 Ca^{2+} bound, an open state (*O*) with 2 Ca^{2+} bound, and an inhibited state (*I*) with 5 Ca^{2+} bound. The rate equations for these four states are given as

$$\frac{dA}{dt} = K_{RA}R + K_{OA}O - (K_{AR} + K_{AO})A,$$

$$\frac{dO}{dt} = K_{IO}I + K_{AO}A - (K_{OI} + K_{OA})O,$$

$$\frac{dI}{dt} = K_{RI}R + K_{OI}O - (K_{IO} + K_{IR})I.$$

The fraction of channels in state *R* is given by the conservation of total probability, i.e.

$$R = 1 - A - O - I.$$

K_{XY} represents the transition rate from state *X* to *Y*. Various transition rates are given as

$$K_{RA} = \left[KR\left(\frac{1}{k_{01}[Ca^{2+}]_i} + \frac{1}{k_{12}[Ca^{2+}]_i^2}\right)\right]^{-1},$$

$$K_{AR} = \left[KA[Ca^{2+}]_i^2\left(\frac{1}{k_{01}[Ca^{2+}]_i} + \frac{1}{k_{12}[Ca^{2+}]_i^2}\right)\right]^{-1},$$

$$K_{AO} = \frac{k_{22}}{KA},$$

$$K_{OA} = \frac{k_{22}}{KO},$$

$$K_{OI} = \left[KO[Ca^{2+}]_i^2\left(\frac{1}{k_{23}[Ca^{2+}]_i^3} + \frac{1}{k_{45}[Ca^{2+}]_i^5}\right)\right]^{-1},$$

$$K_{IO} = \left[KI[Ca^{2+}]_i^5\left(\frac{1}{k_{23}[Ca^{2+}]_i^3} + \frac{1}{k_{45}[Ca^{2+}]_i^5}\right)\right]^{-1},$$

$$K_{RI} = \left[KR\left(\frac{1}{\tilde{k}_{01}[Ca^{2+}]_i} + \frac{1}{\tilde{k}_{45}[Ca^{2+}]_i^5}\right)\right]^{-1},$$

$$K_{IR} = \left[KI[Ca^{2+}]_i^5\left(\frac{1}{\tilde{k}_{01}[Ca^{2+}]_i} + \frac{1}{\tilde{k}_{45}[Ca^{2+}]_i^5}\right)\right]^{-1}.$$

KR, *KA*, *KO*, and *KI* are the occupancy parameters of state *R*, *A*, *O*, and *I* respectively with *KR =1* and

$$KA = \frac{a_1 a_3^{a_2}}{[IP_3]^{a_2} + a_3^{a_2}} + a_4,$$

$$KI = \frac{a_5 a_7^{a_6}}{[IP_3]^{a_6} + a_7^{a_6}} + a_8,$$

$$KO = \frac{a_9 [IP_3]^{a_{10}}}{[IP_3]^{a_{10}} + a_{11}^{a_{10}}}.$$

Various constant involved in the IP_3R model are given in [2].

Converting Ca^{2+}-bound dye to fluorescence units

TIRF microscope measures changes in $[Ca^{2+}]_i$ in terms of fluorescence changes ($\Delta F/F_O$) as Ca^{2+} binds and unbinds to indicator dye Fluo-Where F_0 and ΔF is background fluorescence and change in fluorescence in response to Ca^{2+} binding to Fluo-Thus, we convert Ca^{2+}-bound dye from concentration units to $\Delta F/F_O$,

which was then used in fitting the model to fluorescence signals from TIRF microscopy.
A single channel opening on average increases the fluorescence of a 1.2μm × 1.2μm area around the channel relative to the background signal by 0.11 ± 0.01 (i.e. $\Delta F/F_0$ = 0.11 ± 0.01) in TIRF microscopy experiments using Fluo-4 [4]. We used this information to convert [Ca^{2+}]-bound [dye] to $\Delta F/F_0$ in the model. We simulate a single channel, placed at the center of a 40μm × 40μm area (equal to the area scanned in the TIRF microscopy experiments in [4] as well as in our experiments) and allow it to open for 20ms (the mean open time of IP_3R). The $[Ca^{2+}]_i$ and [dye] equations described above are modified to include diffusion of these two species with the widely accepted diffusion coefficients of 223$\mu m^2/s$ and 200$\mu m^2/s$ for free Ca^{2+} (D_{Ca}) and Fluo-4 (D_{dye}) respectively. Furthermore, to side-step the requirement of using extremely small spatial grid and stay consistent with the spatial resolution of 0.3μm of the TIRF microscopy experiments in [4], we partially adopt the procedure in [1], which simulates the Ca^{2+} release and uptake at the microdomain around the channel separately from the rest of the simulation area. The movement of Ca^{2+} from the microdomain to the nearest grid point (the central grid point in our case) and vice versa is given by diffusion. With these modifications, the equation for $[Ca^{2+}]_i$ becomes

$$\frac{d[Ca^{2+}]_i}{dt} = D_{Ca}\nabla^2[Ca^{2+}]_i + J_{diff} + J_{ipr} + J_{leak} - J_s + k^r_{dye}\big(B_{dye} - [dye]\big) - k^f_{dye}[Ca^{2+}]_i[dye].$$

Where the first and second terms represent the diffusion of Ca^{2+} between neighboring grid points and the transfer of Ca^{2+} from the microdomain around IP_3R to the grid point at the center of simulating area respectively. The functional form of J_{diff} is adopted from [1] where it is modeled with a function similar to Fick's first law, that is, $J_{diff} = k_{diff}([Ca^{2+}]_b - [Ca^{2+}]_i)$. For all other grid points, $J_{diff} = 0$. $[Ca^{2+}]_b$ is the Ca^{2+} concentration in the microdomain and is given by the rate equation

$$\frac{d[Ca^{2+}]_b}{dt} = \gamma_1(J_{ipr} - J_{diff}).$$

J_{ipr} for the single channel is governed by the current through the channel, i.e.

$$J_{ipr} = \frac{1}{2\times F\times \delta V}.$$

Where *I = 0.05pA* is the observed current through IP_3R [5], F is Faraday's constant, and δVis the volume of a hemisphere over the channel with radius of 10nm [6, 7].

With the inclusion of the microdomain in the model, the expression for $[Ca^{2+}]_{ER}$ changes accordingly:

$$[Ca^{2+}]_{ER} = \gamma([Ca^{2+}]_t - [Ca^{2+}]_i\text{-}[Ca^{2+}]_b/\gamma_1).$$

With the inclusion of diffusion, the rate equation for [*dye*] changes to

$$\frac{d[dye]}{dt} = D_{dye}\nabla^2[dye] + k^r_{dye}\big(B_{dye} - [dye]\big) - k^f_{dye}[Ca^{2+}]_i[dye].$$

The above equations were simulated using forward difference method and the peak change in Ca^{2+}-bound dye ($\Delta[dyeCa^{2+}]$) with respect to resting level ($[dyeCa^{2+}]_0$) was recorded, where $[dyeCa^{2+}] = B_{dye} - [dye]$. The ratio $\Delta[dyeCa^{2+}]/[dyeCa^{2+}]_0$ averaged over 1.2μm × 1.2μm area around the channel together with the experimentally observed mean $\Delta F/F_0$ during a single IP_3R opening was used to convert Ca^{2+}-bound dye to $\Delta F/F_0$.

Estimating IP_3 production due to brain extracts

Injection of brain extracts into the oocytes stimulates IP_3 production through a G-protein coupled mechanism. IP_3 activates IP_3R on the ER membrane, releasing Ca^{2+} into the cytoplasm. The global $[Ca^{2+}]_i$ is then represented by $\Delta F/F_0$, imaged through TIRF microscopy. As shown in the main text, after injecting brain extracts, oocytes show a range of fluorescence responses. Guided by the time-trace representing the average fluorescence response in a cell, we write an arbitrary function for $[IP_3]$ ($f[IP_3]$) that loosely resembles the fluorescence traces. For example, for the trace in Fig. 7B (main text), we choose the function $f[IP_3]$ to be

$$f[IP_3] = p_1 \left[\frac{1}{1+exp\left(\frac{p_2-t}{p_3}\right)}\right] exp\left(-\frac{t}{p_4}\right),$$

Where t is time in seconds starting from the injection of brain extract and $p_1 - p_4$ are arbitrary parameters.

Next, the whole-cell model together with $f[IP_3]$ and Ca^{2+}-bound [dye] to $\Delta F/F_0$ conversion) is fitted to the fluorescence trace, optimizing the parameters in $f[IP_3]$ so that the whole-cell Ca^{2+} signaling model gives the best fit to the fluorescence traces. The optimizing in performed in Matlab by computing the least squares error (χ^2) as follows.

The Matlab code first solves the rate equations for the whole-cell and uses the conversion factor for Ca^{2+}-bound dye to $\Delta F/F_0$ to get the whole-cell fluorescence signal given by the model ($\Delta F/F_{o,M}$). Using the whole-cell fluorescence signal from TIRF microscopy experiments ($\Delta F/F_{o,E}$) as observable, we can write χ^2 as

$$\chi^2 = \frac{1}{N}\sum_{k=1}^{N}\left[\left(\frac{\Delta F}{F_{o,M}}\right)_k - \left(\frac{\Delta F}{F_{o,E}}\right)_k\right]^2,$$

where N is the number of data points in the experimental traces. Various parameters in $f[IP_3]$) are determined by minimizing χ^2 for whole-cell TIRF signals. The same procedure is repeated for all oocytes with extracts from different brains.

Mitochondrial function model

The rate equations modeling mitochondrial function are adopted from our previous work [2, 8, 9] and are described in [2] in detail. These equations are originally based on the model in [10]. The model includes processes such as the tricarboxylic acid cycle, electron transport chain, Ca^{2+} signaling pathways, and reactive oxygen species (ROS) production. The time traces of cytosolic Ca^{2+} estimated above are coupled with the mitochondrial model through the rate equation for mitochondrial Ca^{2+} concentration ($[Ca^{2+}]_m$) to investigate changes in cell's ATP and ROS levels due to brain extracts injection. Increase in $[Ca^{2+}]$m stimulates the production of ATP through the tricarboxylic acid cycle, decreases ATP production by depolarizing mitochondrial membrane, and increases ROS production through electron transport chain. Similarly, increase in cytosolic Ca^{2+} causes a decrease in cell's ATP as base cytosolic Ca^{2+} level is restored by ATP-consuming processes.

Numerical and Experimental Methods

Simulations in section "Converting Ca^{2+}-bound dye to fluorescence units" were performed using forward difference method with a time-step of 50µs and a spatial grid size of 0.3µm, equal to the pixel size in the experiments in [4] that estimated the mean $\Delta F/F_0$ value during a single channel opening. Reducing the grid size and time-step did not change our estimates significantly. The initial value of $[Ca^{2+}]_i$ was set to 50nM, approximately equal to the resting cytosolic Ca^{2+} concentration. Based on our simulations, we observed that in resting state approximately 1µM of fluorescence dye was bound to Ca^{2+}. Thus, we set the initial value of [dye] = 39µM. The boundaries were fixed accordingly at steady state values of $[Ca^{2+}]_i$ = 50nM and [dye] = 39µM.

Since there was no stimulus at the start of the experiments, IP_3Rs were initially considered to be in the resting state, that is, the initial values of R, A, O, and I were set at 1, 0, 0, and 0 respectively. In all cases, simulations were allowed to reach steady state before applying any stimulus, so selecting slightly different initial conditions would not change the final results.

To fit the whole-cell model to the TIRF signals, the rate equations were solved in Matlab using the in-built function "ode15s". χ^2 function was minimized using the in-built Matlab function "fminsearch". Using other Matlab optimization functions such as "lsqcurvefit", "fmincon", or "nlinfit" did not change the quality of fits.

Numerical integration of the full model equations (cytosolic Ca^{2+} dynamics, IP3Rs, mitochondrial Ca^{2+} dynamics and bioenergetics) was performed with Intel Fortran compiler (Intel Corporation, Santa Clara, CA). ODEs were solved using RK4 method. Code producing key results in the paper is available upon request from the authors.

Supplementary References

1. Cao, P., et al., *A deterministic model predicts the properties of stochastic calcium oscillations in airway smooth muscle cells.* PLoS computational biology, 2014. **10**(8): p. e1003783.
2. Toglia, P., et al., *Impaired mitochondrial function due to familial Alzheimer's disease-causing presenilins mutants via Ca2+ disruptions.* Cell calcium, 2016. **59**(5): p. 240-250.
3. Ullah, G., et al., *Multi-scale data-driven modeling and observation of calcium puffs.* Cell calcium, 2012. **52**(2): p. 152-160.
4. Smith, I.F. and I. Parker, *Imaging the quantal substructure of single IP3R channel activity during Ca2+ puffs in intact mammalian cells.* Proceedings of the National Academy of Sciences, 2009. **106**(15): p. 6404-6409.
5. Vais, H., et al., *Permeant calcium ion feed-through regulation of single inositol 1, 4, 5-trisphosphate receptor channel gating.* The Journal of general physiology, 2012. **140**(6): p. 697-716.
6. Taylor, C.W., P.C. Da Fonseca, and E.P. Morris, *IP3 receptors: the search for structure.* Trends in biochemical sciences, 2004. **29**(4): p. 210-219.
7. Foskett, J.K., et al., *Inositol trisphosphate receptor Ca2+ release channels.* Physiological reviews, 2007. **87**(2): p. 593-658.
8. Toglia, P., et al., *Data-driven modeling of mitochondrial dysfunction in Alzheimer's disease.* Cell calcium, 2018. **76**: p. 23-35.
9. Toglia, P. and G. Ullah, *The gain-of-function enhancement of IP3-receptor channel gating by familial Alzheimer's disease-linked presenilin mutants increases the open probability of mitochondrial permeability transition pore.* Cell calcium, 2016. **60**(1): p. 13-24.
10. Cortassa, S., et al., *An integrated model of cardiac mitochondrial energy metabolism and calcium dynamics.* Biophysical journal, 2003. **84**(4): p. 2734-2755.
